# An Intelligence Method for Recognizing Multiple Defects in Rail

**DOI:** 10.3390/s21238108

**Published:** 2021-12-03

**Authors:** Fei Deng, Shu-Qing Li, Xi-Ran Zhang, Lin Zhao, Ji-Bing Huang, Cheng Zhou

**Affiliations:** School of Electrical and Electronic Engineering, Shang Hai Institute of Technology, Shanghai 201418, China; 196131105@mail.sit.edu.cn (S.-Q.L.); 206102111@mail.sit.edu.cn (X.-R.Z.); 206101139@mail.sit.edu.cn (L.Z.); 196102104@mail.sit.edu.cn (J.-B.H.); 18656017843@163.com (C.Z.)

**Keywords:** ultrasonic guided waves, principal component analysis, defect recognition, nondestructive testing, multi-signal combination

## Abstract

Ultrasonic guided waves are sensitive to many different types of defects and have been studied for defect recognition in rail. However, most fault recognition algorithms need to extract features from the time domain, frequency domain, or time-frequency domain based on experience or professional knowledge. This paper proposes a new method for identifying many different types of rail defects. The segment principal components analysis (S-PCA) is developed to extract characteristics from signals collected by sensors located at different positions. Then, the Support Vector Machine (SVM) model is used to identify different defects depending on the features extracted. Combining simulations and experiments of the rails with different kinds of defects are established to verify the effectiveness of the proposed defect identification techniques, such as crack, corrosion, and transverse crack under the shelling. There are nine channels of the excitation-reception to acquire guided wave detection signals. The results show that the defect classification accuracy rates are 96.29% and 96.15% for combining multiple signals, such as the method of single-point excitation and multi-point reception, or the method of multi-point excitation and reception at a single point.

## 1. Introduction

As an infrastructure, the structural health of the rails has attracted much attention in the fields of engineering and NDT. Because of the influence of the manufacturing process, the operating situation, and the geographic conditions, rails are prone to various defects. Based on the analysis of the operating situation, rolling contact is the main reason for the rail surface crack. In the manufacturing process, the inclusion in the railhead can lead to an area-shaped section within the rail, which will lead to the formation of transverse cracks under the shelling. Moreover, the natural status, such as air pollution, natural rainfall, and temperature change, provides favorable conditions for the generation of corrosion. In the case of in-service rail, the expansion speed of defects will increase as the size of the defect increases, and the expansion speed is different for different types of defects [1,2,3]. Therefore, further research determining the type and size of rail defects is necessary to ensure proper and effective maintenance and replacement. Attracted by multi-mode and low attenuation, ultrasonic guided waves can perform nondestructive testing of multiple types of defects in long-range rails [4,5,6,7,8]. For instance, the vertical vibration mode is sensitive to cracks at the bottom of rail [9]; the SH mode can detect a transverse defect in rail [8]; the flexural mode can measure axial stress to monitor rail breakage [10]; Evans et al. [11] used ultrasonic guided waves to detect defects in rail level crossings. Lee et al. [12] presented a hybrid analytical-FEM technique based on the dispersion characteristics of the guided wave to design the sensor which can excite specific modes and frequencies for identifying transverse cracking under the shelling. Xing et al. [13] constructed a mathematical model composed of a modal vibration factor and a modal orthogonal factor to select the guided wave modal with the highest sensitivity to detect rail cracks. According to the variation regularity of dispersion characteristics along the longitudinal direction, a three-dimensional dispersion surface with cross-section position information was proposed by Chen et al. [14] for damage detection in turnout straight switch rail. Based on the Lamb wave, Deng et al. [15] proposed the method of automatically searching defects to achieve defect location in the plate structure.

Influenced by the mode of vibration and wavelength, ultrasonic guided waves display different sensitivity to different rail defects. These will induce changes in signal features [16,17,18]. Various signal processing techniques are used to extract signal features from the time, frequency, or time-frequency domain in the field of NDT. Power Spectral Density (PSD) is used to retrieve frequency domain information. The short-term Fourier transformation (STFT) can reflect the characteristics of the time-frequency domain. Researchers extracted various features, including maximum, peak power frequency (PPF), median power frequency (MPF), and STFT coefficients from the original signals, and used an SVM classifier to realize 4 kinds of corrosion defects recognition in the rail foot [19]. Jiang et al. [20] used laser ultrasound to detect defects of different depths on the rail surface. The information generated by the wave-packet transformation, including time-frequency coefficients, local entropy, and energy from the collected detection signals, are used for identifying different defects. Zhou et al. [21] proposed that extracting features by data-driven was used for recognizing defects. Furthermore, it is based on empirical analysis that six features from different domains are extracted from the detection signal. According to the experience, Li et al. [22] extracted six features from the time domain and frequency domain of the guided wave detection signal to identify rail cracks. Torkamani et al. [23] introduced an innovative time-domain damage index named Normalized Correlation Moment (NCM) for identifying laminated composites based on the guided wave. The result was shown to have significant advantages over the signal difference coefficient (SDC), including sensitivity to attenuation of the signal and decreased sensitivity to signal noise. Luca et al. [24] calculated damage indexes (DI’s) to quantify the variations of the signal amplitude caused by the induced damage and chose the DI’s for the Probability Ellipse (PE) method to estimate the probability of the presence of the damage. All the above methods can identify defects. However, these all require professional knowledge or experience to extract the damage index reflecting the defect from the guided wave detection signal to identify the defects.

**Figure 1:**
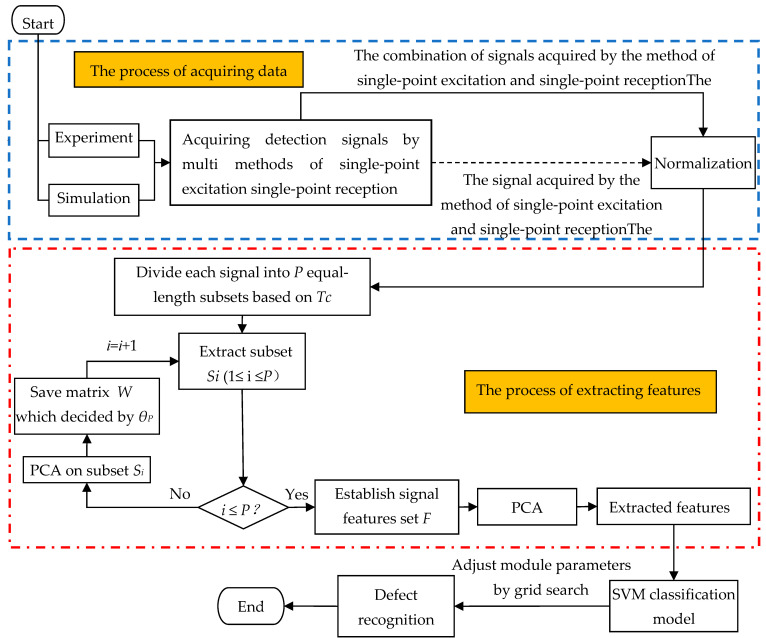
Flow chart of the segmented principal components analysis (S-PCA) for rail defect identification. The enclosed area with the dashed line represents the process of acquiring data; the enclosed area with the dotted line represents the process of extracting features by S-PCA, and the rest area represents the process of classification. *S_i_* is the *i*th sub-signal of the origin signal. And *W* is the matrix of correlation coefficient obtained via PCA with the threshold *θ_P_*.

This paper proposes a new method for identifying rail defects to get away from the limitation of professional knowledge and experience. It can distinguish the defects by combining multiple signals and extracting the features by the S-PCA. The process of identifying rail weaknesses is shown in Figure 1. First, guided wave detection signals are obtained through experiments and simulations of several single-excitation and single-reception methods, as shown in the area enclosed by the round dash line. Then, the S-PCA is used to extract features from the obtained signals, as shown in the area enclosed by the round dotted line. Finally, the SVM classification model enables one to qualitatively and quantitatively identify rail defects based on the extracted features. In signal processing, the restriction of professional knowledge is avoided by dividing the guided wave detection signal and extracting features from each segment. Compared with PCA, S-PCA can dig more weak damage information from guided wave detection signals, which is more conducive to defect identification.

## 2. Materials and Methods

### 2.1. Signal Dividing

The guided wave detection signal is a typical nonstationary time-series signal. The existent defects may cause modal conversion and dispersion of the guided wave signal, and the most intuitive effect is the change of the wave shape. The diverse defect information may be retained in the local zone of the guided wave detection signal. Therefore, we consider that extracting features from each local area realizes the feature extraction of the detection signal. The dividing is an effective means that the feature extraction of the high-dimensional signal is converted into the feature extraction of *P* low-dimensional signals.

(1) $$\left\{\begin{array}{c} Tc=\frac{L}{V_{p}}=\frac{K}{f}\\ P=\mathrm{floor}(\frac{T}{T_{c}}) \end{array}\right.$$

**Figure 2:**
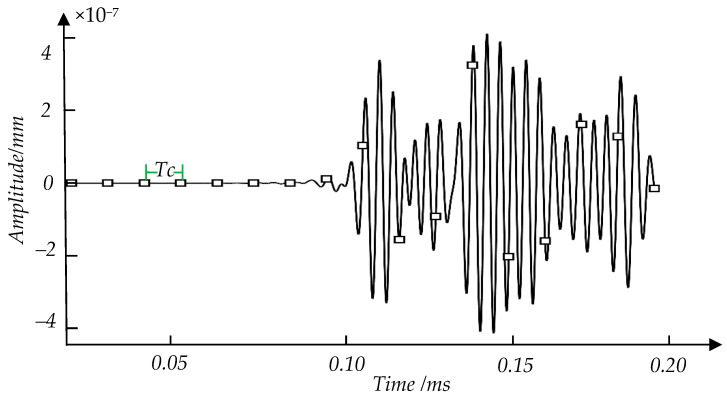
Diagram of the signal divided by *Tc* (12.5 μs) or *L* (5 mm), in which the black boxes represent the dividing points. *Tc* is the time interval between two adjacent dividing points. The vertical axis is the signal amplitude. The horizontal axis is the sampling time of the signal.

*L* is a parament in signal segmentation and is determined by both *L_min_* and *N*. The special expression is *L = N* × *L_min_*, in which *L_min_* is the smallest size of the defect detected by the guided wave selected. *N* is a constant that needs to be determined experimentally. The choice of *N* or *L* will be discussed in the following section. *Tc,* as shown in Equation (1), is equal to the time interval between two adjacent dividing points. *V_P_* is the guided wave group velocity. Figure 2 is a schematic diagram of dividing signals. The guided wave detection signal of crack defect is divided by *Tc* (12.5 μs) or *L* (5 mm).

### 2.2. Principal Component Analysis

PCA is a common method to reduce data dimensions. Its theme is to use low-dimensional data to reflect the valuable information contained in high-dimensional data based on the mapping. Gottumukka et al. [25] used a modular PCA method to improve face recognition technology. Senneville B et al. [26] applied PCA to the motion estimation of abdominal organs. Mazzeo et al. [27] combined wavelet transformed with PCA for the preprocessing of bolts image.

The role of PCA for rail defect recognition is manifested in two aspects: removing redundant information and extracting features. The specific implementation steps are as follows:

- Construct a sample set X of the rail damage characteristics. Matrix A is composed of guided wave detection signals of different defect types or the same type of defects with different levels of damage. As shown in Equation (2), *m* is the number of detection signals, and *n* is the number of features in each sample; $a_{m}$ represents the *mth* detected signal sample with *n* data, and $a_{m1}$ represents the first data in the *mth* sample.
  (2) $$A={\left[\begin{array}{c} a_{1}\\ a_{2}\\ \vdots \\ a_{m} \end{array}\right]}_{m\times 1}={\left[\begin{array}{cc} \begin{array}{cc} a_{11} & a_{12}\\ a_{21} & a_{22} \end{array} & \begin{array}{cc} \cdots & a_{1n}\\ \cdots & a_{2n} \end{array}\\ \begin{array}{cc} \vdots & \vdots \\ a_{m1} & a_{m2} \end{array} & \begin{array}{cc} \ddots & \vdots \\ \cdots & a_{\mathrm{mn}} \end{array} \end{array}\right]}_{m\times n}$$
  The set of samples X of the rail damage characteristic is obtained after centralized processing A. The centralization is expressed in Equation (3), which $\bar{A}$ represents the average value of each column in *A*.
  (3) $$X=A-\bar{A}$$
- Construct the damage covariance matrix *C*. The correlation coefficient in guided wave detection signals can be used to characterize different defects. A covariance matrix is an efficient tool for characterizing the correlation coefficient. The covariance matrix *C* is described by Equation (4).
  (4) $$C=\frac{1}{m-1}X^{T}X$$
- Calculate the eigenvalues and eigenvectors of the damage covariance matrix *C*. According to the matrix decomposition method, the eigenvalues and corresponding eigenvectors of matrix C are solved. Then the eigenvalues are ranked in descending order, $\lambda_{1}\geq \lambda_{2}\geq \ldots \geq \lambda_{n}$, and the corresponding eigenvectors in sequential order, $x_{1}\;x_{2}\ldots \;x_{n}$. Among them, each eigenvector represents a principal component.
- Determine the number of principal components *K*. The information percentage of a principal component is an important reference when the number of principal components is determined. The ratio of one eigenvalue to the sum of all eigenvalues is the information percentage of this principal component, as shown in Equation (5).
  (5) $$\theta_{i}=\frac{\lambda_{i}}{\sum_{i=1}^{n}\lambda_{i}}$$
  Set a threshold *θ* (0 < *θ* < 1) and accumulate the information percentages of the principal components sorted in sequence. When the cumulative sum of the information percentages of the *K*th principal component is greater than or equal to *θ*, there is selected for the *K* principal components.
  (6) $$\theta \geq \frac{\sum_{i=1}^{K}\lambda_{i}}{\sum_{i=1}^{n}\lambda_{i}}$$
- Extract features. The *K* principal components extracted from the covariance matrix *C* are gathered to form a local weight matrix *W*. The process of feature extraction is shown as Equation (7), where *X*’ is a feature set extracted.
  (7) $$X^{\prime }=XW$$

### 2.3. Build S-PCA

**Figure 3:**
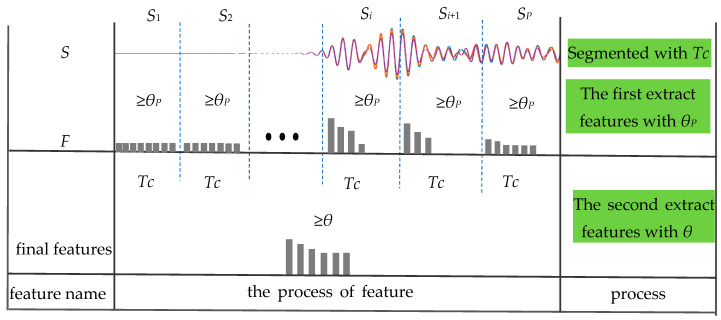
Diagram of segmented principal component analysis of ultrasonic guided waves. *S* is the original signal; *F* is a feature set that combines features from each segment. *S_i_* represents the *i*th subsection; *Tc* is the time interval used for dividing S; *θ_P_* is a threshold used for extracting features from each segment; *θ* is a threshold used for extracting features from *F*. The grey chip represents a feature.

Figure 3 is a schematic diagram of the S-PCA. There are two steps based on the S-PCA to extract features. The first step is to divide. According to the time interval *Tc*, the detection signal *S* is divided into *P* segments of equal length. *S_i_* represents the *i*th signal segment (1 ≤ *i* ≤ *P*). The second step is feature extraction in two sub-steps. The first sub-step is extracting features from each segment by PCA with a threshold *θ_P_* (0 < *θ_P_* < 1) to form a feature set named *F*. The second sub-step is to get the final features from *F* by PCA with a threshold *θ* (0 < *θ* < 1).

### 2.4. SVM Classification Model

SVM realizes classification by searching for the optimal hyperplane determined by a certain number of support vectors. It is often used for solving linearly separable problems. For nonlinear separable problems, the introduction of the kernel function maps the sample data to a high-dimensional space, making it a linearly separable problem, which is then solved by linear classification. The Gaussian radial basis function (RBF) has excellent characteristics of nonlinearity and continuity, so it is often used as the kernel function of SVM. Equation (8) is the expression of RBF, in which *g* is the kernel size of the RBF, and *X* and *X_i_* are feature vectors with the same dimension.

(8) $$\mathrm{Kernel}\left(X,X_{i}\right)=\exp\left(\frac{{\left\|X-X_{i}\right\|}^{2}}{2g^{2}}\right)$$

### 2.5. Classification Model Evaluation

Evaluating the performance of the model provides a basis for the credibility of the model classification results. These are called respectively the precision rate and the recall rate, which are usually used for evaluating the model performance. The precision rate reflects the ability of the model to detect positive samples from the sample set, its expression is shown in Equation (9). The recall rate reflects the ability of the model to detect the number of correctly classified positive samples from all positive samples and is expressed as Equation (10). This article also introduces the F1-score to balance the precision rate and the recall rate to make the model evaluation more accurate, as shown in Equation (11). All the equations above, True Positive (TP) is the number of samples whose labels and the predictions are positive samples. False Positive (FP) is the number of samples where the object labels are negative and the predicted labels inverted. True Negative (TN) is the number of samples whose object labels are positive and the predicted labels inverse. False Negative (FN) is the number of samples whose labels of the object and predicted are negative samples.

(9) $$P=\frac{\mathrm{TP}}{\mathrm{TP}+\mathrm{FP}}$$

(10) $$R=\frac{\mathrm{TP}}{\mathrm{TP}+\mathrm{FN}}$$

(11) $$F1=\frac{2\times P\times R}{P+R}$$

## 3. Experimental Setup

There are 7 different degrees of crack, 5 different degrees of transverse crack under the shelling, and 4 different degrees of corrosion within the railhead. The detect signals of the above defects are acquired by combining the experiments and the simulations.

### 3.1. Experimental Setup

**Figure 4:**
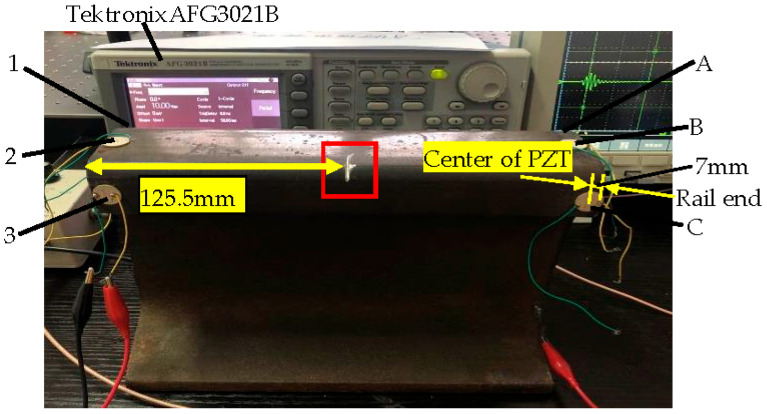
The experimental system for using ultrasonic guided waves detection, in which the position of the red box represents the location of the crack. The PZTs are located at the position of 1, 2, 3, A, B, C, respectively. The distance between the center of PZT and the rail end is 7 mm.

**Figure 5:**
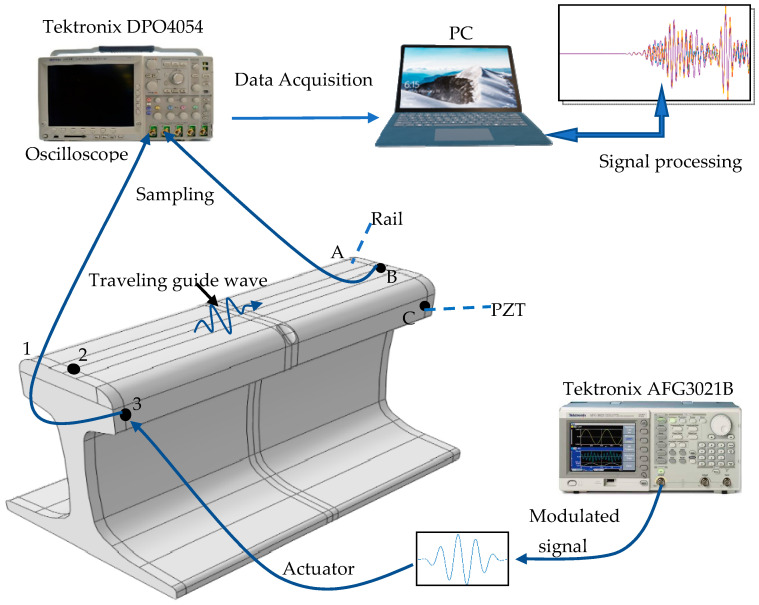
The connection diagram of the detection equipment, in which the Tektronix AFG3021B is the arbitrary function generator and the oscilloscope is used to sample the signals. The PZTs are coupled via ultrasonic gel with the rail surface.

The experimental system is shown in Figure 4. The experimental object is a 60 kg/m rail with a length of 250 mm. Ceramic piezoelectric sheets (PZT-5H, diameter 14 mm, thickness 1 mm, center frequency 200 kHz) are arranged symmetrically on the railhead and marked in sequence as 1, 2, 3, A, B, C. The distance between the center of the piezoelectric films and the rail end is 7 mm. The vertical distance between the rail tread and the center of the piezoelectric films 1, 3, A, or C is 23.57 mm. The piezoelectric plates at positions 1, 2, and 3 are used to excite guided wave signals, and the piezoelectric plates at positions A, B, and C are used to receive detection signals. Any exciting sensors are paired with any receiving sensors to form a signal acquisition channel. There are 9 channels for acquiring signals, like 1TA, 1TB, 1TC, 2TA, 2TB, 2TC, 3TA, 3TB, 3TC. Figure 5 is a connection diagram of the detection equipment. The arbitrary function generator (TektronixAFG3021B) can modulate a sinusoid exciting signal of 200 kHz and 5 circles loaded alternately on piezoelectric slices 1, 2, or 3. The guided wave signal will propagate in the rail. The detection signals are collected using piezoelectric slices A, B, or C. Both the excitation signal and the reception signal are input into the oscilloscope (Tektronix DPO4054) with a specific sampling frequency.

As shown in Figure 4, the location of the crack is indicated by the red square. The crack is artificially cut in the fillet radius on one side of the railhead, located 125.5 mm from the rail end. The crack depth gradually increases at an interval of 1 mm to reflect the varying degrees of crack damage. In this study, 8 kinds of crack defects with different damage levels, 0 mm, 1 mm, 2 mm, 3 mm, 4 mm, 5 mm, 6 mm, 7 mm, are considered, and the corresponding sample labels are 1, 2, 3, 4, 5, 6, 7, 8, respectively.

In the experiment, uncertain physical factors are inevitable, such as the tightness of coupling between the piezoelectric sheet and the surface of the rail. Hence the experiments are repeated 10 times under each excitation-reception mode for each degree of crack to reduce the effect of tightness. The detection signal is picked up at a sampling frequency of 100 MHz, and 10 samples are taken accordingly for each crack degree. 80 samples may be obtained in each signal acquisition mode, and a total of 720 may be obtained from 9 signal acquisition channels. Table 1 shows the types of cracks and the number of samples taken through the experiments of 1TA.

### 3.2. Numerical Simulation

**Figure 6:**
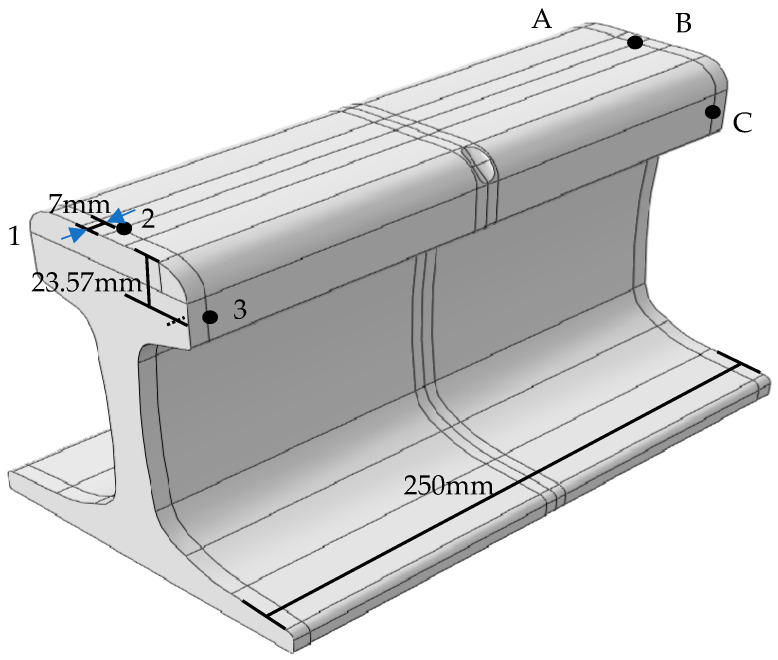
Sensor layouts in the rail simulation model, in which the black dots represent the PZTs.

The finite element simulation software ABAQUS is used to build a model consistent with the experimental object. Table 2 shows the material parameters of the rail model. *ρ* represents density, *E* represents the Elastic Modules, and *υ* represents Poisson’s ratio. The rail model is divided by grid cells C3D8, and the grid size is 1.5 mm. The arrangement of the piezoelectric sheets on the railhead is consistent with the experiment, as shown in Figure 6. The total time to analyze the model is 2 ms, and the time step is 0.01 μs.

**Figure 7:**
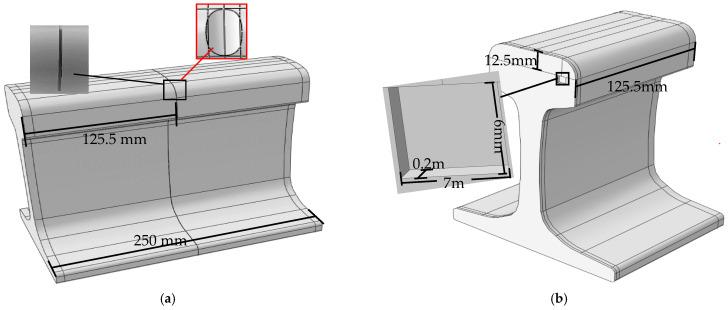
Rail defect model diagram. (**a**) describes the position of the crack and corrosion defect model where the black sequence is the position of the defect, such as corrosion and crack; (**b**) describes the position of the transverse crack under the shelling model.

Figure 7a is the model of rail defects made by ABAQUS, such as crack, corrosion. The position of the black box corresponds to the location of the failure. The method of fine-tuning the defect parameters is adopted to make the numerical simulation and the experimental effect more consistent. Moreover, more detect signals are collected. For crack, the depth of the defect is continuously adjusted with a deviation of 0.001 mm. Taking a 1 mm sample as an example, the crack depth of 0.999 mm, 0.998 mm, 1.001 mm, and 1.002 mm, etc., and these defects are classed as 1 mm cracks. According to the above method, 10 samples are expanded under each excitation-reception mode, there are a total of 80 samples expanded for 8 damage categories. In practice, the depth of crack defect is always irregular. The author uses rounding to make the mark of defects in integers. For instance, the depth of crack less than 1.5 mm carries a 1 mm tag, and the depth of crack which equals 1.5 mm or more carries a 2 mm tag. If the difference between the marked value and the defect depth is within 0.2 mm, the defect depth is indicated with the marked value. For example, the crack depth of 1.2 mm, 1.1 mm, 0.9 mm, and 0.8 mm are all classified as 1 mm cracks. The type of sample expansion is only in the crack depth of 1 mm and 7 mm in this study, and the total number of expanded samples is 58, as shown in Table 3. With respect to corrosion, four defect models of different damage levels, 6 mm^2^, 12 mm^2^, 25 mm^2^ and 30 mm^2^, are constructed by ABAQUS. And the corresponding samples are marked as 9, 10, 11, and 12, respectively. For each type of corrosion defect, the corrosion size is modified by 0.004 mm^2^ to produce more samples. 80 samples are obtained per the excitation-reception method. The transverse crack under shelling is also an area defect. One rectangle is used to approximate the shape of the internal transverse crack. Figure 7b is a rail model with a 6 mm × 7 mm transverse crack under the shelling defect constructed by ABAQUS. 6 mm is the defect’s width, and 7 mm is the length of the defect. The defect is located at a rail length of 125.5 mm and a depth of 12.5 mm from the rail tread. Five transverse cracks under shelling models with different damage levels, 2 mm × 3 mm, 3 mm × 4 mm, 4 mm × 5 mm, 5 mm × 6 mm, 6 mm × 7 mm, are constructed through numerical simulation. Moreover, sample labels corresponding to defect sizes are marked as 13, 14, 15, 16, 17. Under each excitation-reception channel, 21 samples are collected under each type of transverse crack under shelling by adjusting the length or width with the deviation of 0.001 mm.

Table 4 shows that the total number of samples obtained by experiments and simulations under the channel of 1TA, and the sample labels corresponding to defects. Subsequently, all samples obtained by 1TA are made into a dataset including samples and the sample labels. Moreover, the dataset is divided into two parts, which randomly take 80% of the dataset as the training set, the remaining 20% as the testing set. The classify model is trained and tested by training set and testing set, respectively. The data obtained from the remaining 8 signal acquisition methods are processed by the same method with 1TA for subsequent rail defect identification.

## 4. Feature Extraction

### 4.1. Signal Analysis

**Figure 8:**
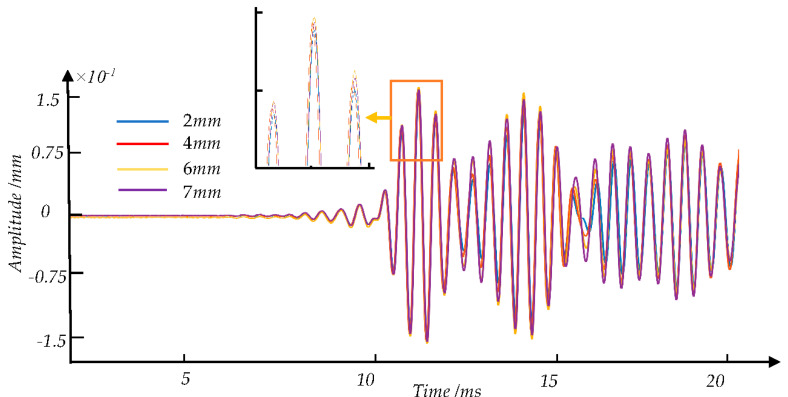
Comparison of experimental signals waveforms of four types of different crack defects with different degrees of damage.

Figure 8 shows the waveform diagram of the crack detection signal obtained at the time range of 0–0.002 s by 2TA in the experiment, and the cracks’ depth is 2 mm, 4 mm, 6 mm, 7 mm, respectively. Based on the guided wave theory, the guided wave group velocity is about 3845 m/s in the experiment. And the speed in the simulation is 3850 m/s, approximately like the result of the experiment. In addition, the experimental signal waveform is like the simulated signal waveform, under different degrees of defect damage. Thus, the simulated signals are used for the expansion of defect samples.

**Figure 9:**
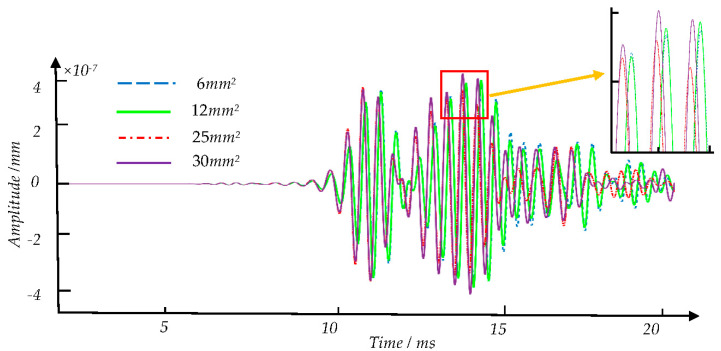
Comparison of the detection signal waveforms of four different corrosion defects with different degrees of damage.

**Figure 10:**
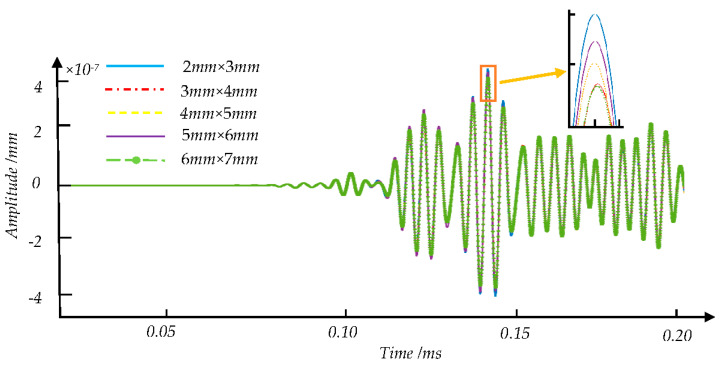
Comparison of five transverse cracks under shelling detection signal waveforms with different levels of damage.

Figure 8, Figure 9 and Figure 10 show the signal waveforms of crack defects, transverse cracks under the shelling, and corrosion defects at different damage levels, respectively. We find that the waveforms of detection signals caused by different damage degrees of defects at the same position are generally similar. According to the elastic wave theory, the corresponding wave packet will cause amplitude changes and overlap due to some factors such as defects. However, the existing method is difficult to distinguish the specific defects by the time domain waveform. In this paper, the amplitude corresponding to each time point on the detection signal is regarded as a feature value in each sample. The statistical analysis and machine learning algorithms are used to find the features between the sample data of defects and correspondence defect types and eliminate redundant features.

### 4.2. Feature Extraction

Feature extraction is a significant part of structural health monitoring (SHM). Redundant features are removed from the detection signal through feature extraction, and several important features are retained to achieve accurate and rapid identification of defects.

*Tc* is an important parameter to achieve feature extraction by S-PCA. And the selection of *Tc* depends on parament *L* and the group velocity *V_P_* of the guided wave signal. In this study, the guided wave group velocity *V_P_* is 3850 m/s. The *L* participating in the discussion is shown in Table 5. *K_P_* is the number of data points in each segment. The influence of different *Tc* on defect identification is stated in Section 6.

PCA is a means of globally reducing data dimensions. Selecting an adequate number of principal components is critical when the PCA algorithm filters the feature. In the process of extracting features from detection signals with S-PCA, *θ_P_* is a threshold to extract principal components from sub-signal segments using PCA. *θ* is a threshold for extracting the principal components from feature set *F*. The influences of these two parameters on rail defect identification are outlined in Section 6, respectively.

## 5. Model Parameter Adjustment

The number of support vectors in the SVM classification model is an essential factor influencing the model performance [28]. The number of support vectors for nonlinear SVM is modified by adjusting the penalty coefficient *C* and the kernel radius *g*. There are always optimal *C* and g values for different datasets to achieve accurate classification of the samples. It is considered that the method of grid search automatically finds suitable C and *g* within the range of [−3, 20], and [−25, 0], for nine datasets.

## 6. Experiment Results and Discuss

### 6.1. Influence of Tc on the Defect Recognition

The guided wave detection signals are divided into *P* sub-signal segments with a time interval *Tc*. To select a suitable *Tc*, we assume that both *θ_P_* and *θ* are equal to 99%. When *Tc* is equal to 0.5 μs, 2.5 μs, 5 μs, 7.5 μs, 10 μs, and 12.5 μs, the accuracy of SVM varies in shown in Table 6.

In Table 6, the red number represents the maximum classification accuracy attained by the classifier based on the selected parameters in each dataset. When *Tc* is 5 μs, seven datasets, such as 1TA, 1TB, 1TC, 2TA, 2TB, 3TA, and 3TB, reach the maximum classification accuracy rate. When *Tc* is equal to 7.5 μs, both 2TC and 3TC reach the maximum classification accuracy rate. But when *Tc* is 5 μs, the classification results of 2TC and 3TC are not much different from the optimal result maintaining a relatively high classification level. According to the content above, most of the nine datasets may achieve the best classification effect when *Tc* is equal to 5 μs. So 5 μs is selected as the optimal parament of *Tc* for subsequent analysis.

### 6.2. Influence of θ_P_ on The Rail Defects Identification

*θ_P_* controls the number of principal components extracted from each sub-signal. Different *θ_P_* can extract different numbers of principal components from the sub-signal segments and affects the classification results of the classification model. It is the initial step that *Tc* is equal to 5 μs and *θ* is equal to 99%. Table 7 shows the variation of the classification results in the nine datasets with *θ_P_* selected in turn from 10%, 20%, 30%, 40%, 50%, 60%, 70%, 80%, 90%, and 99%.

As shown in Table 7, when *θ_P_* equals 50%, any one of 1TB, 1TC, 2TA, 2TB, 2TC, 3TA, and 3TC reaches the maximum classification accuracy. For 1TB, when *θ_P_* is equal to 60%, the classification accuracy is the same as the optimal classification result. However, when *θ_P_* equals 50%, the number of the features used to classify defects is 5. When *θ_P_* equals 60%, the number is 7. Thus 50% is taken at 1TB to reduce the loss of computation. When *θ_P_* equals 60%, 1TA and 3TB reach the best classification outcomes at 91.95% and 94.25%, respectively. For 1TC and 2TC, when *θ_P_* equals 10%, they attain the maximum classification accuracy. Moreover, when *θ_P_* is equal to 20%, 30%, 40%, and 50%, in turn, the classification accuracy keeps the same classification result equaled to the optimal result. However, when *θ_P_* equals 10%, 20%, 30%, and 40%, in turn, the 13th category cannot be identified in 3TB, as shown in Table 8. The reason is that the value of *θ_P_* is too low to extract enough principal components. When *θ_P_* equals 50%, all categories can be recognized, and when *θ_P_* equals 60%, the classification accuracy rate is 94.25% that is the best-classified outcome for 3TB. According to the analysis above, most of the nine datasets can maintain a relatively high classification accuracy when *θ_P_* equals 50%. Thus 50% is selected as the optimal parament of *θ_P_* for subsequent research.

### 6.3. Influence of θ on the Rail Defect Identification

*θ* is an important parameter to extract features from *F* which are final features. Table 9 shows that when *Tc* equals 5 μs and *θ_P_* equals 50%, the classification results of the nine datasets vary with *θ* changed from 10%, 20%, 30%, 40%, 50%, 60%, 70%, 80%, 90%, and 99%. According to Table 9, the classification accuracy rates of the nine datasets show an increasing trend as the *θ* value increases. Therefore, 99% is chosen as a fixed parameter of *θ* for follow-up research.

In summary, 5 μs, 50%, and 99% are selected, respectively, as the optimal parameters, which are the fixed values of *Tc*, *θ_P_*, *θ*.

### 6.4. Classification Result of Single-Channel Acquisition Signal

The signals collected by different signal acquisition channels are turned into corresponding datasets. Once each dataset is normalized, the S-PCA model with selected paraments is utilized to extract features. The dataset is split into the training set and the testing set. The outcome of the classification is the average of the 10 results of the classification. The variance *S* represents the standard deviation of 10 classification results, which is used for evaluating the stability of the classification. And the results of nine datasets are presented in Table 10.

Table 10 shows that among the 9 signal acquisition methods, the accuracy rates of the SVM classification model exceed 76% that is a medium score. In 1TA, 1TB, 2TA, 2TB, 2TC, and 3TB, the SVM classification accuracy rates reach nearly or more than 90%, which is a good score. Both 2TA and 2TB have excellent classification because the sensor at position 2 can be smoothly attached to the track surface, making the defect information in the collected detection signal more obvious. For 3TB, the distance between the sensor and the defect is relatively close, which makes the difference of the defect signals more prominent, which is conducive to the identification of the defect. Both 1TA and 1TB have higher classification accuracy because the signal reflected by the defect can be received by the A and B sensors, which is conducive to the recognition defect. In addition, the S-PCA is an effective method of extracting features and can extract the local differences in the detection signal, which is useful for the classifier to make the correct distinction. Similarly, the distance between the exciting sensor and the receiving sensor is relatively long, so the classification results of 1TC and 3TA are 76.15% and 76.53%, respectively, lower than the signals collected by other signal acquisition methods.

### 6.5. Classification Result of Multi-Channel Signal Combination

The above research shows that a single channel collecting signal can identify different types and degrees of defects damage on the railhead. Based on theoretical analysis, a single-channel acquisition signal can only reflect part of the characteristics of the defect. Thus, 1TC and 3TA classification accuracy rates are lower. The two types of combination methods are comprehensively analyzed to explore the influence of multi-channel signals on rail defect recognition, such as multi-point excitation and single-point reception or single-point excitation and multi-point reception. The types of combinations and the corresponding classification results are indicated in Table 11 and Table 12.

Table 11 shows the results of the classification of the combined methods of single-point excitation and multi-points reception signals. With the number of combined signals increasing, it is found that the classification accuracy rate shows an increasing trend, such as 1TAC, 1TBC, and 1TABC; 2TAB, 2TAC, 2TBC, and 2TABC; 3TAB, 3TAC, 3TBC, and 3TABC. Moreover, compared to the classification of signals collected by one single channel, the standard deviation *S* of the classification is slightly reduced after multiple signal combinations. The above results show that the accuracy of the classification can be effectively enhanced by the combination method. Since the propagation path of each signal is different, the effective combination can more widely reflect the health status of the detected object. The same results can also be found in Table 12.

## 7. Conclusions

Aiming to identify multiple defects in rail, experiments and numerical simulations are used to focus on the ultrasonic guided wave detection signals of crack defect, transverse crack under shelling, and corrosion defect. By modifying and combining the sensor positions, a sample library of defect detection signals is obtained by 9 different signal excitation-reception methods. The S-PCA algorithm is proposed to extract the features of the signal to eliminate the dependency on professional knowledge. Furthermore, the extracting features are input into the SVM classifier to identify the type and extent of defects. At the end of the research, the following conclusions are drawn:

- The method of extracting features from the segments of detection signal by PCA can effectively eliminate redundant information in the signal and retain adequate information, which improves the accuracy of quantitative and qualitative identification of rail defects.
- The detection signals collected from different excitation-reception positions describe the overall health of the different parts of the rail. Obtaining the combined detection signal through single-point excitation and multi-point reception or multi-point excitation and single-point reception can more comprehensively describe the health status of the detection object, which is good to improve the accuracy of defect recognition.
- The S-PCA algorithm is an efficient method of extracting features based on statistical theory. It does not rely too much on the professional knowledge of guided wave detection, which reduces the difficulty of rail defect identification. Furthermore, the method could be more easily implemented in practical engineering in the future.

## Figures and Tables

**Table 1:** The types of cracks and the quantities of samples taken from the experiments of exciting at position 1 and receiving the signal at position A.

Damage Degree	Number of Samples	Damage Degree	Number of Samples
intact	10	4 mm	10
1 mm	10	5 mm	10
2 mm	10	6 mm	10
3 mm	10	7 mm	10

**Table 2:** Configuring the rail model built by *ABAQUS*.

Model	Length	*ρ*	*E*	*υ*
60 kg/m	250 mm	7.85 × 10^−9^	2 × 10^5^	0.29

**Table 3:** The type of crack and the quantity of samples collected in a simulation of exciting at position 1 and receiving the signal at position A.

Defection Type	Defection Category (mm)	Total Number of Samples	Actual Damage (mm)	Number of Samples
intact	intact	10	0	10
crack	1	30	1.0	10
			1.1	10
			1.2	10
	2	30	1.8	10
			1.9	10
			2.0	10
	3	10	3.0	10
	4	10	4.0	10
	5	10	5	10
	6	10	6	10
	7	28	6.9	6
			7	10
			7.1	6
			7.2	6

**Table 4:** Sample type and the total number of samples collected by exciting at the position of 1 and receiving the signal at position A.

Defection Type	Sample Label	Defection Category	Number of Samples	Total Number of Samples
intact	1	-	20	403
Crack defect	2	1 mm	40	
	3	2 mm	40	
	4	3 mm	20	
	5	4 mm	20	
	6	5 mm	20	
	7	6 mm	20	
	8	7 mm	38	
Corrosion defect	9	6 mm^2^	20	
	10	12 mm^2^	20	
	11	25 mm^2^	20	
	12	30 mm^2^	20	
Internal nuclear defect	13	2 mm × 3 mm	21	
	14	3 mm × 4 mm	21	
	15	4 mm × 5 mm	21	
	16	5 mm × 6 mm	21	
	17	6 mm × 7 mm	21	

**Table 5:** Selection of the parameter *L* and time interval *Tc*.

N	L(mm)	*Tc* (μs)	K_p_	P
2	0.2	0.5	50	400
10	1.0	2.5	250	80
20	2.0	5.0	500	40
30	3.0	7.5	750	27
40	4.0	10.0	1000	20
50	5.0	12.5	1250	16

**Table 6:** The influence of *Tc* on the accuracy of defect classifications, in which the red number is the best-classified result in this dataset.

	0.5 μs	2.5 μs	5.0 μs	7.5 μs	10.0 μs	12.5 μs
1TA	86.21	86.21	87.36	86.21	86.21	86.21
1TB	94.25	95.40	95.40	90.80	91.95	90.80
1TC	77.01	77.01	79.31	78.16	79.31	78.16
2TA	94.25	95.40	95.40	95.40	95.40	93.10
2TB	90.80	90.80	93.10	91.95	89.66	88.51
2TC	88.51	88.51	88.51	89.66	89.66	88.51
3TA	72.41	74.71	75.86	73.56	72.41	72.41
3TB	89.66	89.66	90.80	89.66	89.66	88.51
3TC	90.81	91.95	91.95	93.10	90.81	91.95

**Table 7:** The effect of *θ_P_* on classification accuracy, in which the red number is the best classification result in this dataset.

	10%	20%	30%	40%	50%	60%	70%	80%	90%	99%
1TA	89.66	89.66	89.66	89.66	90.80	91.95	90.80	89.66	89.66	88.51
1TB	88.51	88.51	88.51	88.51	94.25	94.25	89.66	90.80	88.51	87.36
1TC	79.31	79.31	79.31	79.31	79.31	77.01	70.11	78.16	75.86	78.16
2TA	95.40	95.40	95.40	95.40	96.55	93.10	94.25	93.10	94.25	90.80
2TB	94.25	94.25	94.25	94.25	96.55	96.55	95.40	95.40	95.40	94.25
2TC	90.80	90.80	90.80	90.80	90.80	88.51	88.51	88.51	88.51	83.91
3TA	72.41	72.41	72.41	72.41	73.56	68.97	68.97	67.82	68.97	67.82
3TB	81.61	81.61	81.61	81.61	89.66	94.25	91.95	87.36	83.91	87.36
3TC	86.21	86.21	86.21	86.21	90.80	89.66	87.36	88.51	89.66	86.21

**Table 8:** 3TB’s confusion matrix when *θ_P_* equals 10%, *Tc* equals 5 μs, and *θ* equals 99%.

	1	2	3	4	5	6	7	8	9	10	11	12	13	14	15	16	17
precision	1.00	0.75	0.75	1.00	1.00	1.00	0.75	0.90	0.75	1.00	0.75	1.00	0	1.00	0.80	0.60	1.00
recall	1.00	0.67	0.75	1.00	1.00	1.00	1.00	0.90	0.75	0.80	1.00	1.00	0	1.00	0.80	0.50	0.71
F1-score	1.00	0.71	0.75	1.00	1.00	1.00	0.86	0.90	0.75	0.89	0.86	1.00	-	1.00	0.80	0.55	0.83

**Table 9:** The influence of θ on classification accuracy in which the red circle is the best classification result in this dataset.

	10%	20%	30%	40%	50%	60%	70%	80%	90%	99%
1TA	49.43	49.43	49.43	49.43	74.71	74.71	74.71	77.01	80.46	90.80
1TB	42.53	42.53	42.53	63.22	63.22	63.22	73.56	73.56	85.06	94.25
1TC	50.57	50.57	50.57	66.67	66.67	66.67	66.67	66.67	71.26	78.16
2TA	54.02	54.02	54.02	75.86	75.86	75.86	83.91	89.66	89.66	96.55
2TB	58.62	58.62	58.62	58.62	82.76	82.76	90.80	90.80	93.10	95.40
2TC	48.28	48.28	48.28	48.28	73.56	73.56	73.56	83.91	89.66	88.51
3TA	42.53	42.53	42.53	42.53	55.17	55.17	55.17	59.77	64.37	73.56
3TB	58.62	58.62	58.62	58.62	81.61	81.61	81.61	85.06	87.36	94.25
3TC	50.57	50.57	50.57	70.11	70.11	74.71	74.71	80.46	78.16	90.80

**Table 10:** The SVM’s classification results of single-channel acquisition signal.

	Accuracy	S	Precision	Recall	F1-Score
1TA	90.99%	0.03	91.20%	90.13%	89.83%
1TB	90.22%	0.02	91.95%	90.07%	90.19%
1TC	76.15%	0.04	82.01%	73.76%	74.43%
2TA	93.63%	0.02	94.12%	93.54%	93.32%
2TB	92.75%	0.02	94.68%	92.36%	92.05%
2TC	90.11%	0.03	90.79%	89.26%	89.06%
3TA	76.53%	0.03	83.10%	79.71%	76.89%
3TB	90.77%	0.02	91.80%	90.26%	90.29%
3TC	88.02%	0.03	89.90%	87.88%	87.43%

**Table 11:** The SVM’s classification results of feature combinations method of single-point excitation and multi-point reception.

Combination Type	Accuracy	S	Precision	Recall	F1-Score
1TAB	96.15%	0.02	96.29%	95.59%	95.51%
1TAC	93.08%	0.01	93.67%	92.37%	92.08%
1TBC	91.21%	0.02	92.56%	90.295	90.50%
1TABC	94.73%	0.02	95.32%	94.00%	94.20%
2TAB	93.74%	0.02	95.42%	93.51%	93.20%
2TAC	94.07%	0.01	94.99%	93.66%	93.58%
2TBC	94.95%	0.02	96.34%	95.10%	94.87%
2TABC	96.15%	0.01	96.63%	96.13%	95.93%
3TAB	89.01%	0.03	90.25%	88.36%	88.51%
3TAC	88.79%	0.02	90.57%	88.49%	87.77%
3TBC	88.90%	0.02	90.62%	88.38%	88.34%
3TABC	90.55%	0.03	91.64%	90.31%	89.99%

**Table 12:** The SVM’s classification results of the combination method of multi-point excitation and single-point reception.

Combination Type	Accuracy	S	Precision	Recall	F1-Score
1TA + 2TA	96.29%	0.02	96.34%	96.06%	95.70%
1TA + 3TA	89.78%	0.01	90.39%	87.82%	87.58%
2TA + 3TA	92.75%	0.02	93.23%	92.72%	92.43%
1TA + 2TA + 3TA	95.49%	0.02	96.12%	95.20%	95.03%
1TB + 2TB	95.38%	0.01	96.32%	95.35%	94.94%
1TB + 3TB	93.08%	0.02	94.54%	92.35%	92.59%
2TB + 3TB	94.03%	0.02	95.31%	94.71%	94.10%
1TB + 2TB + 3TB	93.41%	0.02	95.23%	93.79%	93.45%
1TC + 2TC	92.09%	0.02	93.02%	92.05%	92.00%
1TC + 3TC	90.22%	0.03	91.97%	89.74%	89.06%
2TC + 3TC	91.43%	0.03	93.35%	90.98%	90.83%
1TC + 2TC + 3TC	92.09%	0.03	93.44%	92.22%	91.95%

## Data Availability

Not applicable.
